# Hypersexual behaviour among young adults in Germany: characteristics and personality correlates

**DOI:** 10.1186/s12888-022-04370-8

**Published:** 2022-12-19

**Authors:** Dennis Jepsen, Petra J. Brzank

**Affiliations:** 1 Institute of Medical Sociology, Martin-Luther-University Halle-Wittenberg, Faculty of Medicine, Magdeburgerstraße 8, 06112 Halle (Saale), Germany; 2 Institute of Social Medicine, Rehabilitation Sciences and Healthcare Research, University of Applied Science Nordhausen, Weinberghof 4, 99734 Nordhausen, Germany

**Keywords:** Compulsive sexual behaviour disorder, Hypersexuality, Problematic pornography use, Sex addiction, Sexual compulsivity, Sexual health, Social Psychiatry, Young adults

## Abstract

**Background:**

Hypersexual behaviour (HB) is characterized by recurring unsuccessful efforts to control intense, repetitive sexual impulses that result in sexual activities and manifest in the behaviour of the concerning individual over an extended period. This study aims to describe the characterization of HB among the target group, identify personality correlates, and associations between HB and a lack of sexual education and reflection during school time.

**Methods:**

A cross-sectional design was used to survey the participants (age 18–27; *n* = 609) online who were recruited via a web forum for addiction selfcare, a website for casual sex dating, Facebook and the mail distribution of the University of Applied Science Nordhausen (Germany). Standardised questionnaires were used to measure the key variables in the study. The sexual behaviour (masturbation, pornography consumption, promiscuity), several personality tendencies, and retrospective information about sexual education of the participants during school time were investigated. Correlation and binomial logistic regression were used to analyse the data with HB as the outcome variable.

**Results:**

10.5% (*n* = 64) of the participants were identified as hypersexual. The assignment to male sex, a problematic pornography consumption, and impulsive tendencies were determined as predictors of HB. Strong correlates were identified between HB and promiscuity, and impairments in important areas of life. Weak to moderate correlates comprise between HB and all investigated personality traits, and several aspects of sexual risk behaviour. No association was found between HB and a lacking sexual education during school time. The descriptive analysis revealed further sexual problems within the sample (e.g. feelings of shame and guilt, sexual risk behaviour, sexual dysfunction). *Conclusion.* Young adults should get more attention as a vulnerable group for the development of hypersexuality in sexual education, prevention and therapy. Regarding to its treatment, HB therefore should be considered together with its associations with sociodemographic information, personality traits, and psychosocial factors.

**Supplementary Information:**

The online version contains supplementary material available at 10.1186/s12888-022-04370-8.

## Background

Hypersexual behaviour (HB) in a clinical understanding is defined as Compulsive sexual behaviour disorder (code: 6C72) in the eleventh revision of the International Classification of Diseases (ICD-11) [1]. It is characterized by recurring unsuccessful efforts to control intense, repetitive sexual impulses that result in sexual activities and manifest in the behaviour of the concerning individual over an extended period (six months is indicated to provide orientation) [2]. The diagnosis further includes “marked distress or significant impairment in personal, family, social, educational, occupational, or other important areas of functioning” [2].

HB can be described by the characteristic excessive masturbation and predominance of autoerotic behaviour, problematic pornography consumption, and promiscuity [3, 4]. In a recently published systematic review by Grubbs et al. a prevalence of HB between 1 and 10% was reported [5], while heterosexual men and LGBTIQ men and women [6] are at a higher risk of demonstrating HB. Thus, the authors emphasize that these findings should be interpreted with caution, because all analysed studies were limited to Western countries, various scales for measuring and definitions of hypersexuality were used (the survey instruments were developed prior to the recently included diagnosis in ICD-11) and the samples are just partially comparable. Although it became clear that there have been no epidemiological studies to elaborate the prevalence of addictive sexual behaviour to date [5], several studies focussing self-reported hypersexual behaviour were conducted within the past years [5, 7–10], mainly addressing problematic pornography consumption.

Scientists assume that in our times, HB cannot be investigated without considering the technological development and establishment of the internet within the last years [5]. At the same time, adolescents are considered as vulnerable group for the development of HB, mainly because a reflexive media literacy and sexual scripts are under development during that life phase [11]. In addition to that, projects promoting sexual health during adolescence are rare in Germany [12], and associated with shame and stigmatization [13]. Taking these insights into account, especially young adults seem an interesting target group for the investigation of HB.

Moreover, numerous studies indicate various psychosocial impairments in relation with HB, and several authors draw attention to the direct consequences of HB on health. Therefore, a generally negative association between HB and mental health has been identified [14]. In general, associations with personal and interpersonal consequences are defined as characteristic for HB, for example, impairment of relationships, school, and work life, as well as financial problems [15]. In addition to that, several studies investigated the relationship between HB and personality traits that can be relevant for the psychotherapeutic treatment of HB. Positive correlations were found between HB and neuroticism [16, 17], hostility [16], sensation seeking [16, 18], impulsivity [18, 19], loneliness and lower levels of satisfaction with life and happiness [15, 20]. Also, a relation between religiosity and HB is suggested, possibly explained by moral incongruence and feelings of shame and guilt regarding the individual sexual behaviour of the affected persons [21, 22]. HB occurs often simultaneously with other psychiatric diseases and is associated with the adherence of them (e.g. ADHD, depression, personality disorders [4, 23], bulimia nervosa, and alcohol, cannabis, or cocaine abuse and addiction [23]). HB is also associated with a dysfunctional family structure [24], experiences of violence, child and adult sexual abuse [25], and related posttraumatic stress disorder among males and females [26]. Further, sexual risk behaviour is another problem that is related to HB [27], which can lead to medical problems (e.g. sexually transmitted diseases).

Hence, HB is related to social and health problems, as well as to therapeutic-relevant personality structures, and thus, a relevant topic for sex education, (social) pedagogy, psychotherapy, and medicine.

## Objectives

A literature research on PubMed, Sage Pub and Springer Link (search items: hypersexuality, hypersexual behaviour, compulsive sexual behaviour, impulsive sexual behaviour, excessive sexual behaviour, sex addiction, problematic pornography consumption) revealed a lack of knowledge about HB among young adults. This cross-sectional study, firstly, aims to identify characteristics of HB in young adults and, secondly, find correlates between HB and personality traits, and associations between HB and a lack of sexual health education during school time. Discovering more about these aspects can reveal needs regarding the prevention and therapy of HB and lead to a development of adequate sexual education and HB prevention programmes during adolescence.

## Methods

### Conduction

This cross-sectional study collected data from young adults (18 to 27 years old) using an online survey (www.soscisurvey.com) in the German language. The eight-week survey period (December 2020 until January 2021) was chosen due to time restrictions concerning the data collection as part of a Master thesis. Possible participants were approached via the web forums *Addiction and Selfcare Forum* (*Sucht- und-Selbsthilfe-Forum)* and *poppen.de*, a dating website for casual sex contacts, where the link to the questionnaire was promoted in their newsfeed and forums. Further participants were collected via Facebook (the link was posted on several Facebook groups promoting scientific online research) and the mail distribution of the University of Applied Science Nordhausen (Germany).

### Questionnaire

All items were phrased with gender-equitable and -sensitive language [21]. The questionnaire was divided into four parts. A survey guide, listing all used items formulated by the authors can be find as supplementary material (Additional file 1).

1. Demographical information, such as age, gender, current place of residence, migrant background, religious affiliation, and highest educational degree. Gender was determined on the basis of a non-binary understanding, while participants were asked about their gender identity with the answer options *female*, *male*, *non-binary*, *other* (with the opportunity of concretization via free text function) and *prefer not to say*. The exact wording of the used items can be found in the supplementary material (Additional file 1, pp. 1–2).
2. Sexual profile included questions about the conditions of relationships and sexual life (e.g. relationship status, sexual orientation, age at the first time of sexual intercourse), items to analyse opportunities to reflect sexual topics, and the presence of sexual education during childhood and youth. The exact wording of the used items can be found in the supplementary material (Additional file 1, pp. 2–3).
3. HB was identified using the Hypersexual Behaviour Inventory (HBI) via the subscales coping (e.g. with distress, dysphoric conditions), consequences (undesired implications of sexual behaviour), and control (deficits in the control of the behaviour) [3, 28]. The scale contains 19 items with answer options of *never* to *very often* on a five-point Likert scale. A summation index variable was constructed with the cut-off value for the prevalence of hypersexuality ≥ 53 [28]. Considering the numerous scales measuring sexual addictive behaviour, and the new diagnostic criteria of compulsive sexual behaviour disorder in ICD-11, the HBI was chosen because 1) it shows high reliability and validity and is well established within hypersexuality research [29], and 2) parallels can be identified between the subscales and the ICD-11 diagnosis criteria (impairments in controlling sexual impulses, the neglect of important areas of life, and individual psychological strain).
  The masturbation behaviour was analysed based on items of frequency of masturbation and the age of the first masturbation. Items of the subscale “reasons” of the Attitudes Towards Masturbation Scale (ATMS) [30] were added to identify compulsive reasons for masturbation via a seven-point Likert scale with options between *no reason* and *very important reason*. The answer options of the Attitudes Towards Masturbation Scale were conformed (1 = *never* to 7 = *always*) to guarantee the consistency of the items used within the survey. Only participants with masturbation experience could answer these questions. Within the supplementary material, it can be reproduced which items of the ATMS were used in the survey (Additional file 1, pp. 3–4).
  The problematic pornography consumption as one character of HB was determined by the Problematic Pornography Consumption Scale (PPCS) by Böthe et al. [31]. The inventory measures a problematic pornography consumption according to the diagnosis criteria of addiction (salience, mood modification, tolerance, withdrawal, conflicts, and relapse) with 18 items. The cut-off value of PPCS prevalence is ≥ 76 [31]. Again, only participants who have already consumed pornographic material could answer these questions.
  A nine-item promiscuity scale (α = 0.857) was generated out of promiscuity-defining characteristics, according to relevant literature [4, 22], e.g. “I would like to have as many different sexual partners as possible” or “The idea of attaching to one specific person in terms of sex makes me feel bored”. The five-point scale ranged from 1 = *fully disagree* to 5 = *fully agree*. The used items to measure promiscuous behaviour are listed in the supplementary material (Additional file 1, p. 5).
  A six-item scale to measure feelings of shame and guilt regarding to masturbation (α = 0.886) was generated out of following items: *After masturbation… I feel shame, I think about what my parents could think of me, I think about what others could think of me I feel indecent/ perverse, I do not feel comfortable,* and *I feel guilty*. The five-point scale ranged from 1 = *fully disagree* to 5 = *fully agree*. The used items to measure feelings of shame and guilt are listed in the supplementary material (Additional file 1, pp. 6–7).
  In addition, sexual risk behaviour and sexual functioning problems were surveyed via respective items. For the exact wording of the items please consider the supplementary material (Additional file 1, pp. 5–6).
4. Personality traits and attitudes: these items were measured via shortened versions of established personality inventories: narcissism (Narcissistic Personality Inventory, NPI) [32], impulsivity (Impulsive Behaviour Scale, I-8) [33], depression (Patient Health Questionnaire, PHQ) [34], low self-esteem (Self-Esteem Scale) [35], and stresses in primary socialization (Biographical Questionnaire for Alcohol Addicted People, BIFA-AL) [36]. Exemplary items to capture impairments in primary socialization are “I did not feel well at home, because we did not have a good family life”, “My mother/ father always criticised me”, and “In my childhood/youth, I was often punished too hard”. Five items were used to measure histrionic tendencies, including characteristics of the cognitive profile of histrionic personality disorder [37]. Characteristics indicating loneliness were investigated with three items of the University of California, Los Angeles Loneliness Scale [38], supplemented with the item “I would describe myself as a loner.” To reproduce, which items of the described inventories were included, and the exact wording of them, please consider the supplementary material (Additional file 1, pp. 7–9).

Finally, participants were asked, whether they had experienced harassment or sexual abuse and had undergone psychotherapy in the past or currently (see supplementary material, Additional file 1, p. 9).

### Statistical analyses

Bivariate analyses were conducted to identify relations between HB and the independent variables (correlation calculation addressing HBI-Score as dependent variable and *χ*^*2*^-tests with *φ*-coefficient calculation addressing the presence of HB as dependent variable according to the HBI cut-off value of ≥ 53 with dichotomous coding). Binomial logistic regression was used to identify predictors of HB, using the presence of HB as dependent variable and including all independent variables based on theoretical considerations (variables that showed statistic associations with HB in previous research or are discussed in relevant literature, see background section). Additionally, we included migration background in the regression model because of its potential associations to traumatic stress [39], which in turn are related to HB. The data analyses were conducted via the software *IBM SPSS Statistics, Version 27*.

## Results

### HB and sociodemographic information

The sample size was *n* = 609 with an average age of *M* = 23.1 (*SD* = 2.7). A total of 58.5% of the participants were assigned to female sex, 40.2% to male sex and 1.0% stated they were non-binary. Further demographic characteristics are presented in Table 1 (appendix). The HBI cut-off value of ≥ 53 was reached by *n* = 64 (10.5%) participants, *n* = 15 of them stated female gender, *n* = 49 male gender assignment.

**Table 1:** Sociodemographic data of the sample

	*n* (*h* in %)					
	Total population (*n* = 609)			Subpopulation classified as hyper-sexual (*n* = 64)		
	Total (*n* = 609)^1^	Female (*n* = 356)	Male (*n* = 245)	Total (*n* = 64)	Female (*n* = 15)	Male (*n* = 49)
***Current place of residence***^***2***^						
Village/country town	139 (22.8)	71 (19.9)	68 (27.8)	15 (23.4)	1 (6.7)	14 (28.6)
Small-sized town	87 (14.3)	58 (16.3)	29 (11.8)	8 (12.5)	2 (13.4)	6 (12.2)
Middle-sized town	216 (35.5)	143 (40.2)	73 (29.8)	15 (23.4)	6 (40.0)	9 (18.4)
Large town	112 (18.4)	61 (17.1)	51 (20.8)	20 (31.3)	4 (26.7)	16 (32.7)
Megapolis	41 (6.7)	20 (5.6)	21 (8.8)	5 (7.8)	2 (13.4)	3 (6.1)
* Missing data*	6 (1.0)	3 (0.8)	3 (1.2)	1 (1.6)	0 (0.0)	1 (2.0)
***Immigration background***						
Yes	123 (20.19)	70 (19.7)	53 (21.6)	15 (23.4)	3 (20.0)	12 (24.5)
No	471 (77.3)	284 (79.8)	187 (76.3)	47 (73.4)	12 (80.0)	35 (71.4)
* Missing data*	7 (1.1)	2 (0.6)	5 (2.0)	2 (3.1)	0 (0.0)	2 (4.1)
***Religious affiliation***						
Buddhist	2 (0.3)	2 (0.6)	0 (0.0)	0 (0.0)	0 (0.0)	0 (0.0)
Evangelic	175 (28.7)	114 (32.0)	61 (24.9)	17 (26.6)	3 (20.0)	14 (28.6)
Hindu	9 (1.5)	0 (0.0)	9 (3.7)	2 (3.1)	0 (0.0)	2 (4.1)
Jewish	0 (0.0)	0 (0.0)	0 (0.0)	0 (0.0)	0 (0.0)	0 (0.0)
Catholic	117 (19.2)	65 (18.3)	52 (21.2)	17 (26.6)	1 (6.7)	16 (32.7)
Muslim	4 (0.7)	1 (0.3)	3 (1.2)	1 (1.6)	0 (0.0)	1 (2.0)
No religious affiliation	215 (35.3)	129 (36.2)	86 (35.1)	20 (31.3)	10 (66.7)	10 (20.4)
Other	16 (2.6)	9 (2.5)	7 (2.9)	1 (1.6)	0 (0.0)	1 (2.0)
* Missing data*	63 (10.3)	36 (10.1)	27 (11.0)	6 (9.4)	1 (6.7)	5 (10.2)
***Highest educational degree***						
Secondary school leaving certificate (9th grade)	9 (1.5)	2 (0.6)	7 (2.9)	3 (4.7)	0 (0.0)	3 (6.1)
Secondary school leaving certificate (10th grade)	27 (4.4)	10 (2.8)	17 (6.9)	9 (14.1)	2 (13.4)	7 (14.3)
General university entrance qualification	313 (51.4)	224 (62.9)	89 (36.3)	22 (34.4)	8 (53.4)	14 (28.6)
Completed vocational training	74 (12.2)	24 (6.7)	50 (20.4)	9 (14.1)	1 (6.7)	8 (16.3)
Bachelor degree	130 (21.3)	78 (21.9)	52 (21.2)	13 (20.3)	3 (20.0)	10 (20.4)
Master degree	28 (4.6)	8 (2.2)	20 (8.2)	6 (9.4)	0 (0.0)	6 (12.2)
Other	15 (2.5)	6 (1.7)	9 (3.7)	2 (3.2)	0 (0.0)	1 (2.0)
No educational qualification	3 (0.5)	3 (0.8)	0 (0.0)	1 (1.6)	1 (6.7)	0 (0.0)
* Missing data*	2 (0.3)	1 (0.3)	1 (0.4)	0 (0.0)	0 (0.0)	0 (0.0)
***Relationship status***						
Single	244 (40.1)	131 (36.8)	113 (46.1)	28 (43.8)	9 (60.0)	19 (38.8)
Monogamous relationship	307 (50.4)	207 (58.1)	100 (40.8)	22 (34.4)	4 (26.7)	18 (36.7)
Open relationship	31 (5.1)	10 (2.8)	21 (8.6)	6 (9.4)	0 (0.0)	6 (12.2)
Polyamorous relationship	7 (1.1)	4 (1.1)	3 (1.2)	2 (3.1)	0 (0.0)	2 (4.0)
Other form of relationship	9 (1.5)	4 (1.1)	5 (2.0)	5 (7.8)	2 (13.4)	3 (6.1)
* Missing data*	3 (0.5)	0 (0.0)	3 (1.2)	1(1.6)	0 (0.0)	1 (2.0)
***Sexual orientation***						
Heterosexual	397 (65.2)	246 (69.1)	151 (61.6)	31 (48.4)	7 (46.7)	24 (49.0)
Homosexual	19 (3.1)	9 (2.5)	10 (4.1)	1 (1.6)	0 (0.0)	1 (2.0)
Bisexual	94 (15.4)	39 (11.0)	55 (22.4)	21 (32.8)	4 (26.7)	17 (34.7)
Pansexual	15 (2.5)	10 (2.8)	5 (2.0)	2 (3.1)	1 (6.7)	1 (2.0)
Asexual	6 (1.0)	4 (1.1)	2 (0.8)	0 (0.0)	0 (0.0)	0 (0.0)
Other	14 (2.3)	8 (2.2)	6 (2.4)	3 (4.7)	1 (6.7)	2 (4.0)
* Missing data*	56 (9.2)	40 (11.2)	16 (6.5)	6 (9.4)	2 (13.4)	4 (8.2)

*Note*: *n* = Absolute frequency, *h* = Relative frequency. ^1^ Participants who stated to be non-binary (n = 6) and participants who answered the item addressing gender assignment with *prefer not to say* (n = 2) were only considered in this column, because they were just a small number. ^2^ Numbers of inhabitants: Village/country town: below 5,000. Small-sized town: between 5,000 and below 20,000. Middle-sized town: between 20,000 and below 100,000. Megapolis: as of 1,000,000

A positive answer to the items *I have received psychotherapeutic/ psychiatric treatment in the past* and/or *Currently I receive psychotherapeutic/ psychiatric treatment* were used to identify respondents who are or have been receiving psychiatric/psychotherapeutic treatment. About one fifth of the entire sample (19.9%, including 3,8% classified as hypersexual) reported that they had already have been in and 5.6% are currently under such kind of treatment (including 1.3% classified as hypersexual), which was not a focussed treatment for HB specifically.

About a third of the entire sample (33.0%) stated former harassment experiences during school time (including 5.7% classified as hypersexual), and 6.7% currently (including 2,0% classified as hypersexual). Experiences of abuse during childhood were reported by 4.1% (including 1.1% classified as hypersexual), and during adulthood by 5.2% (including 1.3% classified as hypersexual).

### HB and general sexual behaviour

The average ages of first masturbation, pornography consumption, and intercourse of the entire population, as well as the mean number of sexual partners, in comparison with the as hypersexual and as not hypersexual classified subgroups are shown in Table 2. Considering t-test calculation there was a significant difference of the average age of first masturbation (*t*(378) = 3.78, *p* < 0.001) and the average age of first pornography consumption (*t*(327) = 4.01, *p* < 0.001) between participants not classified as hypersexual and participants classified as hypersexual, no respective significant difference was identified related to average age of first intercourse (*t*(417) = 1.19, *p* = 0.235). Another significant difference was found between the mean number of sexual partners of participants not classified as hypersexual and participants classified as hypersexual (*t*(354) =—3.75, *p* < 0.001). Two statistical outliers were identified regarding the number of sexual partners, which were excluded from the calculation of mean and t-test.

**Table 2:** Means of first sexual experiences stratified by the classification as hypersexual

	***M (SD)***		
	Total population (*n* = 609)	Subpopulation not classified as hypersexual (*n* = 545)	Subpopulation classified as hypersexual (*n* = 64)
Average age of first masturbation	13.4 (2.95)	13.7 (2.92)	12.2 (2.99)
Average age of first pornography consumption	14.4 (3.03)	14.7 (3.06)	12.8 (2.86)
Average age of first intercourse	16.7 (2.50)	16.7 (2.49)	16.3 (2.76)
Mean number of sexual partners	5.7 (5.00)	5.4 (4.92)	8.7 (5.44)

*Note: M* Mean, *SD* Standard deviance; *n* = absolute frequency

Comparisons of the frequency distribution of individuals classified as hypersexual and the entire population regarding to problems in sexual activity and sexual risk behaviour are shown in Tables 3 and 4. In addition to that, 7.1% of the entire sample state that they had already been infected by a sexually transmitted disease; another 4.1% were not sure whether their infection was sexually transmitted. Feelings of guilt (25.0%) and shame (21.8%) due to the individual execution of sexual activities were also found within the population.

**Table 3:** Frequency of problems in sexual activity of individuals classified as hypersexual in comparison with the entire population

	***n* (*h* in %)**	
	Total population (*n* = 609)	Subpopulation classified as hypersexual (*n* = 64)
Premature orgasm	101 (16.5)	20 (31.2)
Absence of orgasm	115 (18.9)	9 (14.1)
Long delays of orgasm	154 (25.3)	15 (23.5)
Absence of sexual desire	45 (7.4)	5 (7.8)
Impairments of sexual arousal	25 (4.1)	6 (9.4)
Pain during sex	18 (3.0)	2 (3.2)

*Note: n* = Absolute frequency, *h* = Relative frequency. Included are participants who answered the item “Please state, how often you experience following problems in sexual activity” with *often, very often* and *always*

**Table 4:** Frequency of sexual risk behaviour of individuals classified as hypersexual in comparison with the entire population

	***n* (*h* in %)**	
	Total population (*n* = 609)	Subpopulation classified as hypersexual (*n* = 64)
Prostitution	47 (7.7)	17 (26.6)
Sex under influence of illegal drugs	133 (21.8)	24 (37.5)
Sexual activities prohibited by law	76 (12.5)	19 (29.7)
Sexual activities, which harmed somebody physically or mentally	54 (8.9)	16 (25.0)

*Note: n* = Absolute frequency, *h* = Relative frequency. Included are participants who answered the item “Please state, how often you performed following sexual activities” with *seldom, occasionally, often, very often* and *always*

Moderate associations were found between the frequency of masturbation and the prevalence of HB (*χ*^*2*^(5) = 59.797, *φ* = 0.353, *p* < 0.001) and with the HBI score (*r*_*s*_ = 0.450, *p* < 0.001). Furthermore, moderate effects were found between HB and compulsive reasons for masturbation, as shown in Table 5. A weak association between feelings of guilt and shame regarding masturbation and the prevalence of HB (r_pb_ = 0.296, *p* < 0.001) and the HBI score (*r*_*s*_ = 0.321, *p* < 0.001) was found. The HBI score correlates strongly with the promiscuity score (*r*_*s*_ = 0.580, *p* < 0.001), while a moderate association was found between the prevalence of hypersexuality and the promiscuity score (r_pb_ = 0.496, *p* < 0.001).

**Table 5:** Associations between HB and compulsive reasons for masturbation

*Below potential reasons for masturbation are listed. Please estimate how often you masturbate for these reasons*	Presence of HB (*χ*^*2*^*; φ*)
If I feel frustrated about something else	*χ*^*2*^*(6)* = 131.500; 0.527***
Because I cannot prevent it, even if I try	*χ*^*2*^*(6)* = 122.320; 0.510***
It’s a compulsive sexual behaviour	*χ*^*2*^*(6)* = 120.379; 0.504***
It’s a habit	*χ*^*2*^*(6)* = 96.508; 0.452***
To avoid cheating on my partner	*χ*^*2*^*(6)* = 55.918; 0.348***
I get aroused by sexual activities that are not socially acceptable, and so I can avoid executing them	*χ*^*2*^*(6)* = 81.001; 0.415***

Note: *** *p* < 0.001. Contrasting the answers through dichotomization (*often, very often,* and *always* = *compulsive reasons present* vs. *never, seldomly, occasionally* and *sometimes* = *compulsive reasons not present)*. HB was dichotomized (*cut-off reached* vs. *cut-off not reached)*

Associations between the presence of HB as dichotomous variable and compulsive reasons for masturbation are shown in Table 5.

Weak to moderate effects were found between the prevalence of HB and the following aspects regarding risky sexual behaviour: Performing sexual actions which are not legally permissible (*χ*^*2*^(4) = 23.878, *φ* = 0.238, *p* < 0.001), performing sexual actions which harmed other people mentally and/or physically (*χ*^*2*^(4) = 27.766, *φ* = 0.256, *p* < 0.001), the offering of sex work (*χ*^*2*^(4) = 25.992, *φ* = 0.248, *p* < 0.001), and sex under the influence of illegal drugs (*χ*^*2*^(4) = 25.436, *φ* = 0.245, *p* < 0.001).

A strong association was found between impairments in important social relationships and the presence of HB (*χ*^*2*^(4) = 130.497, *φ* = 0.555, *p* < 0.001), and the HBI score (*r*_*s*_ = 0.514, *p* < 0.001). A moderate association remained between financial problems and the presence of HB (*χ*^*2*^(4) = 76.164, *φ* = 0.397, *p* < 0.001) and HBI score (*r*_*s*_ = 0.338, *p* < 0.001). No associations were identified between HB and the presence of sexual functioning problems.

### Personality correlates

Weak and moderate associations were found between the presence of hypersexuality or HBI score and all personality traits investigated (see the results in Table 6). No association was found between the presence of HB and current or former harassment, sexual abuse, or a participation in psychotherapeutic treatment.

**Table 6:** Associations between HB and personality traits

Personality trait^1^	Presence of HB^2^ (χ^2^; φ)	HBI score (r_s)_
Impulsivity	*χ*^*2*^*(17)* = 76.169; 0.397***	0.377***
Narcissism	*χ*^*2*^*(31)* = 36.540; 0.275	0.338***
Histrionic tendencies	*χ*^*2*^*(24)* = 38.362; 0.282*	0.307***
Depression	*χ*^*2*^*(12)* = 28.580; 0.243**	0.295***
Low self-esteem	*χ*^*2*^*(12)* = 28.580; 0.243**	0.191***
Loneliness	*χ*^*2*^*(18)* = 56.142; 0.341***	0.272***
Stresses in primary socialization	*χ*^*2*^*(17)* = 76.169; 0.397***	0.262***

*Note:* *** *p* < 0.001. ^1^ Participants who reached a mean score of ≥ 3.5 on the related personality scales were measured. ^2^ The presence of HB was dichotomized with 1 = *HBI cut-off reached* and 0 = *HBI cut-off not reached*

### HB and sex education during school time

Only 30.5% of the participants stated that they were educated about everything they wanted to know about sexuality at school. Solely 15.8% reported that there were personnel for open dialogues on sexuality-related topics, and 13.7% that there were educational offers addressing the various facets of sexuality. No association was found between HB and a self-stated lack of sexual education during school time.

### Predictors of HB

The results of binomial logistic regression with the presence of HB (dichotomous coding, score ≥ 53) as an independent variable are shown in Table 7. Male sex assignment (*B* = 1.254, *OR* = 3.505, *95% CI* [1.127, 10.895], *p* = 0.030), a problematic pornography consumption (*B* = 3.803, *OR* = 44.853, *95% CI* [7.794, 258.123], *p* < 0.001), and impulsive personality tendencies (*B* = 2.974, *OR* = 19.567; *95% CI* [4.796, 79.821], *p* < 0.001) were identified as significant predictors of HB. The binomial logistic regression model was statistically significant, *χ*^*2*^ (20) = 113.572, *p* < 0.001. The amount of explained variance (Nagelkerkes *R*^*2*^) was large, *R*^*2*^ = 0.578. The overall percentage of accuracy in classification was 91.9% with a sensitivity of 62.2% and a specificity of 97.8%.

**Table 7:** Results of binomial logistic regression with the presence of HB as an independent variable

Categorial variable	*B*	*p*	*OR*	*95% CI*
*Sociodemographic information*				
Gender assignment *(male)*	**1.254**	**0.030**	**3.505**	**[1.127, 10.895]**
Immigration background *(yes)*	-0.133	0.810	0.875	[0.296, 2.590]
Religious affiliation *(religious)*	0.324	0.539	1.382	[0.492, 3.885]
Relationship status *(single)*	0.810	0.180	2.249	[0.688, 7.345]
Sexual orientation *(heterosexual)*	0.361	0.488	1.435	[0.517, 3.983]
*Personality correlates*^1^				
Low self-esteem *(yes)*	-0.954	0.613	0.385	[0.010, 15.497]
Loneliness *(yes)*	2.487	0.068	12.029	[0.833, 173.769]
Narcissism *(yes)*	0.558	0.450	1.748	[0.411, 7.436]
Depression *(yes)*	-0.073	0.937	0.929	[0.152, 5.678]
Histrionic tendencies *(yes)*	0.833	0.230	2.301	[0.590, 8.974]
Impulsivity *(yes)*	**2.974**	***	**19.567**	**[4.796, 79.821]**
Impairments in primary socialization *(yes)*	1.404	0.090	4.073	[0.804, 20.622]
*Hypersexual behaviour*				
Problematic pornography consumption *(yes)*^2^	**3.803**	***	**44.853**	**[7.794, 258.123]**
Promiscuous behaviour *(yes)*^3^	0.980	0.319	2.664	[0.388, 18.306]
*Dysfunctional psychosocial experiences*				
Victim of bullying in the past *(yes)*	-0.333	0.554	0.716	[0.237, 2.163]
Victim of bullying in the present *(yes)*	0.897	0.419	2.453	[0.279, 21.567]
Victim of sexual abuse as a minor *(yes)*	1.992	0.301	7.334	[0.168, 319.502]
Victim of sexual abuse as an adult *(yes)*	-0.180	0.870	0.836	[0.097, 7.197]
Formerly under psychotherapeutic treatment *(yes)*	-0.769	0.227	0.464	[0.133, 1.614]
Currently under psychotherapeutic treatment *(yes)*	0.358	0.750	1.430	[0.158, 12.900]
**Constant**	-23.366	***	0.000	

*Note:* Significant predictors are shown in bold. ^1^ Participants who reached a mean score of ≥ 3.5 on the related personality scales were classified. ^2^ Participants who reached a score of ≥ 76 on the PPCS were classified. ^3^ Participants who reached a mean score of ≥ 3.5 on the promiscuity scale were classified. *** *p* < 0.001

## Discussion

### Characteristics of HB among young adults

All in all, many of the findings regarding characteristics of HB among young adults in Germany match closely with the results of previous research on hypersexuality in general. Consistent with prior studies [5], in this study, HB seems to occur far more often in male than in female young adults. To the best of our knowledge, so far these gender-based differences have not yet been addressed in research and need further investigation, particularly when considering that the symptom structure of HB in men and women is similar [40]. Focussing on biological (e.g., endocrinal system, neurological processes), and social aspects of gender (e.g., role expectations, attachment experiences), it seems beneficial to explore this relationship in greater detail.

Furthermore, earlier first sexual experiences (earlier firstpornography use, and intercourse) seem to be related to the presence of HB (consistent with Engel et al. 2019, [27]). Adolescents are considered particularly vulnerable to developing sexual problems because sexual experiences within a too early stage of sexual development can lead to difficulties in processing sexual cues and thus to the development of sexual problems like HB [41]. In combination with that, the found strong association between HB and problematic pornography consumption suggests a prominent role of digital sexual media in the manifestation of HB. This association underlines the importance to consider pornography consumption when reflecting on hypersexual behaviours. One possible explanation for the mentioned difficulties in the sexual life of young adults is the strong relation between sexuality and digitalization, and the assumed influences on intimate relationships [41]. These phenomena should get more space in further research of clinically relevant sexual behaviour. No matter how diverse the investigated sample of young adults in Germany might be, they do have one thing in common: they all grew up during a peak of digitalization and individualization. Sexuality and the creation of a sexual identity constitute important areas of life and development [42], which are closely connected to these dominating cultural phenomena [5, 11].

We also found another characteristic of HB among young adults in Germany, i.e., several compulsive reasons for masturbation, which can describe the expression of HB in more detail, regarding to the symptom of the loss of control included in the diagnosis of compulsive sexual behaviour disorder.

Furthermore, the strong associations between HB and risky sexual behaviour seem to be particularly interesting since it has not yet been addressed in previous research. According to this, the results further reveal indications of delinquent behaviour tendencies, as associations between HB and the performance of sexual actions that are not legally permissible or which harmed other people mentally and/ or physically were identified. However, we did not assess in detail what was understood of delinquent behaviour tendencies concretely. Further research is needed to investigate which role this kind of behaviour play in the forensic perspective on HB, as well as in connection with legal problems as consequences of HB, building on previous research [15, 43]. In addition, the revealed association between HB and sex under influence of illegal drugs support results within chemsex research, which outline potential risks for health, like infection with sexually transmitted diseases, and the development of depression, substance addiction, and drug-induced psychosis [44]. Consequently, illegal sexual behaviour and sexual risk behaviour are accompanied by HB and can lead to several consequences for health and existential problems.

### Correlates between HB and personality

Particularly strong effects were found between HB and impulsive tendencies, as has been already stated in previous research [15, 18, 29]. Although not all analyses of the relation between stresses in primary socialisation and the presence of HB were significant, the associations were strong and support previous findings of a dysfunctional family structure as an important risk factor for the development of HB [24, 45]. Interestingly seem the associations between narcissistic and histrionic tendencies, which should be considered in the psychotherapeutic treatment of HB as well as appropriate personality disorders.

### Associations between HB and a lack of sexual education during school

Although no association was found between HB and a lack of sexual education during school time, sexual education seems scarce among young adults in Germany. Sexual risk behaviour in the form of sex under the influence of illegal drugs and sexual activities that harm other people physically or mentally were stated by many participants, as well as feelings of guilt and shame in relation to sexuality, no matter whether they were classified as hypersexual or not. Further indications of current and former psychosocial impairments within the general population (e.g., harassment, sexual abuse) were identified in a serious proportion of participants. These results of the descriptive analysis portray a sobering balance concerning the distribution of demand and supply addressing the reflection of individual sexuality for young adults in Germany. Regarding the consequences of HB, the development of preventive measures addressing these problems seem beneficial. Therefore, schools especially represent an important setting where sexual education programmes should be included, because the earlier maladaptive patterns of sexual behaviour are identified, the better they can be worked on and realigned in a beneficial manner [46]. Therefore, skilled personnel should create spaces for reflection in the form of project days or workshops for students in which different facets of sexuality are discussed – not just biological basics and safer sex (as is currently operated in Germany). Discretion should play an important role, thus, an opportunity for bilateral conversations with school psychologists, school social workers, or appropriate externals should be established.

### Limitations and strengths

The HBI is an instrument for self-assessment. However, the diagnosis of compulsive sexual disorder cannot be made without an evaluation by medical specialists and/or psychotherapists [29]. Consequently, whether hypersexuality is prevalent within the population according to a clinical understanding is not clear. Furthermore, shortened versions of personality inventories were used, meaning that information about their validity and reliability cannot be transmitted from relevant literature. Although the male to female ratio in the participants is nearly balanced, very few women were classified as hypersexual, so concrete assertions about HB in women specifically are not fully possible and results of gender comparisons cannot be conducted with the expectation of valid results. For the description of HB especially in women, publications by Klein et al. [47], Kowalewska et al. [18], and Montgomery-Graham [48] should be considered. Moreover, it is questionable whether the construct of HB pursued in HBI can be operationalized for women and men equally. While interpreting the results, it should be considered that the participants were approached on a website for self-identified addicted persons and a dating website for casual sex contacts, which could bias the selection of the sample. Moreover, we do not have any information how many participants were approached via which online platform, so we cannot estimate the extent of the suspected bias. Although HBI is validated for the German [49], Italian [50], and Spanish population [51], it is questionable whether the results of this study are generalizable for other countries as well, considering the cultural differences regarding definitions of sexual health. Hence, further research with randomized and larger samples is necessary to reach external validity and enable international comparability.

One clear strength of the study is the diversity regarding the distribution of gender and sexual orientation within the sample. The gender ratio is nearly balanced, while the proportion of queer people (25.4%) is high in comparison to results of a current survey of sexual orientation in Germany [52]. Furthermore, the inventories used for measuring hypersexuality (HBI; [28]) and a problematic pornography consumption (PPCS; [31]) show high reliability and validity and are well established within hypersexuality research. The self-generated scale for promiscuity shows a very good reliability. Because HBI and Kafka’s diagnosis criteria were used to measure HB, the results of this study can be compared to the many other studies that have used these instruments as well. In addition, various data were collected that, to this date, had not been subjects of previous research on HB (e.g. associations with, sexual education, reasons for masturbation, or sexual functioning problems). To the best of our knowledge, associations between HB and narcissistic and histrionic tendencies were surveyed last over twenty years ago with smaller sample sizes [53, 54]. Further, in this study we could survey more facets of sexual risk behaviour in relation with HB, while the focus of previous research was on unprotected sexual intercourse and intercourse with multiple sexual partners [55]. Thus, the results of this study are an important tile which can elucidate and supplement the mosaic of hypersexuality research.

## Conclusion

This study gives an overview of therapeutic-relevant personality traits, behaviour, and experiences which are related to HB among young adults in Germany. 10.5% (*n* = 64) of the participants were identified as hypersexual. The assignment to male sex, a problematic pornography consumption, and impulsive tendencies were determined as predictors of HB. Strong correlates were identified between HB and promiscuity, and impairments in important areas of life. Weak to moderate correlates comprise between HB and all investigated personality traits, and several aspects of sexual risk behaviour. No association was found between HB and a lacking sexual education during school time. The descriptive analysis revealed further sexual problems within the sample (e.g. feelings of shame and guilt, sexual risk behaviour, sexual dysfunction). More research is needed to examine, how these aspects are linked together. Young adults should get more attention as a vulnerable group for the development of hypersexuality in sexual education, prevention and therapy. Regarding its treatment, HB should therefore be considered together with its associations with sociodemographic information, personality traits, and psychosocial factors. Hence, institutions for sexual education, counselling, and therapy are faced with several challenges to provide purposeful, comprehensive, and individually adapted psychosocial care and the prevention of HB. Thus, negative consequences in social and vocational life, the development of comorbidities, sexual risk behaviour, and the aggravation of the addictive pathology can be prevented with an early diagnosis. Regarding to its treatment, HB therefore should be considered together with its associations about sociodemographic information, personality traits, and psychosocial factors.

## Supplementary Information

**Additional file 1**.

## Data Availability

The participants were ensured, that the information collected would be processed solely within the scope of the research project and not shared with third parties. Thus, the datasets generated and/or analysed during the current study are not publicly available but are available from the corresponding author on reasonable request.

## Abbreviations

HB: Hypersexual behaviour
HBI: Hypersexual Behaviour Inventory
ICD-11: Eleventh revision of the International Classification of Diseases
PPCS: Problematic Pornography Consumption Scale
