# Supplementary material for: Hypersexual behaviour among young adults in Germany: characteristics and personality correlates
Source: BMC Psychiatry. 2022 Dec 19;22:804. doi: 10.1186/s12888-022-04370-8 (PMC9762014; doi:10.1186/s12888-022-04370-8)
Supplement: Supplementary file 1 — Additional file 1. [file 12888_2022_4370_MOESM1_ESM.docx]

**Hypersexual behaviour among young adults in Germany – Characteristics and personality correlates**

**Survey guide**

Dennis Jepsen • Petra J. Brzank

**Contact**

Dennis Jepsen, M.A. Therapeutic Social Work

Institute of Medical Sociology

Martin-Luther-University Halle-Wittenberg, Faculty of Medicine

Magdeburgerstraße 8

06112 Halle (Saale) - Germany

E-Mail: dennis.jepsen@medizin.uni-halle.de

**Question block 1: Socio-demographic Information**

- Age

*Answer options: Please enter [Free text input]; Prefer not to say*

- Gender assignment

*Answer options: Female; Male; Non-binary; Other [Free text input]; Prefer not to say*

- Current place of residence

*Answer options: Village/country town (below 5,000 inhabitants); Small-sized town (between 5,000 and below 20,000 inhabitants); Middle-sized town (between 20,000 and below 100,000 inhabitants); Large town (as of 100,000 inhabitants); Megapolis (as of 1,000,000 inhabitants); Prefer not to say*

- Immigration background

*Answer options: Yes; No; Prefer not to say*

- Religious affiliation

*Answer options: Buddhist; Evangelic; Hindu; Jewish; Catholic; Muslim; No religious affiliation; Other [Free text input]; Prefer not to say*

- Highest educational degree

*Answer options: Secondary school leaving certificate (9th grade); Secondary school leaving certificate (10th grade); General university entrance qualification; Completed vocational training; Bachelor degree; Master degree; Other [Free text input]; No educational qualification*

**Question block 2: Sexual profile**

General information about sexual profile

- Relationship status

*Answer options: Single; Monogamous relationship; Open relationship; Polyamorous relationship; Other form of relationship [Free text input]; Prefer not to say*

- If you were to describe your sexual orientation with one of the following terms, which would you choose?

*Answer options: Heterosexual; Homosexual; Bisexual; Pansexual; Asexual; Other [Free text input]; Prefer not to say*

- How old were you when you had sex for the first time?

*Answer options: Please enter age [Free text input]; I do not know; I have never had sex; Prefer not to say*

- How old were you when you first masturbated?

*Answer options: Please enter age [Free text input]; I do not know; I have never masturbated; Prefer not to say*

- How old were you when you watched porn for the first time?

*Answer options: Please enter age [Free text input]; I do not know; I have never masturbated; Prefer not to say*

Sexual education during schooltime

*Answer options:* *Strongly disagree; Rather disagree; Partly agree; Rather agree; Strongly agree*

- I was educated about everything I wanted to know about sexuality when I was in school.
- There was the possibility in my school to talk openly about questions concerning sexuality (e.g., with social pedagogues, school psychologists, liaison teachers).
- I was able to talk openly with my parents about sexuality during my youth and ask questions about it.
- There were regular programs at my school that dealt with various facets of sexuality.
- I had the opportunity in my youth to talk to other people about my personal sexuality.

**Question block 3: Problematic sexual behaviour**

Hypersexual behaviour was identified using the German version of the Hypersexual Behaviour Inventory (HBI) by Klein et al. [1]. The problematic pornography consumption as one character of hypersexual behaviour was determined by the Problematic Pornography Consumption Scale (PPCS) by Böthe et al. [2]. The German version of the scale was provided by Beata Böthe (University of Montreal, Canada) and Johannes Fuss (University of Duisburg-Essen, Germany).

(Compulsive) reasons for masturbation

*Following the Attitudes Toward Masturbation Scale by Young & Muehlenhard [3].*

You can see below a list of potential reasons to masturbate. Please estimate how often you masturbate for these reasons.

*Answer options: Never; Seldom; Occasionally; Sometimes; Often; Very often; Always; Prefer not to say*

- Because I find it pleasurable.
- If I am not getting as much sex as I want.
- If I am bored.
- To relieve stress.
- Because – even though I try – I cannot stop myself.
- To learn how to have better orgasms.
- It is a good way to take a break (e.g., break from studying).
- If I am so sexually aroused that it is interfering with other things I want or need to do.
- So that I can focus my concentration on a task after masturbation.
- To help me fall asleep.
- It is a habit.
- If I feel frustrated about something else.
- To avoid using another person for sex.
- I just do it without really thinking about it.
- It is a compulsive sexual behaviour.
- I get aroused by sexual activities that are not socially acceptable, so I can fantasize about them during masturbation.
- To learn how to give myself pleasure.
- To make myself a better partner.
- *(Added item)* To avoid cheating on my partner.

Promiscuity

Please estimate to which extent the following statements apply to you.

*Answer options: Strongly disagree; Rather disagree; Partly agree; Rather agree; Strongly agree*

- Anonymity is important to me during sex.
- As soon as one of my sexual partners bores me, I start searching for new sexual contacts.
- I would like to have as many different sexual partners as possible.
- My goal is to conquer/win around potential sexual partners.
- Whether I consider a person to be attractive does not play a role in my choice of sexual partners.
- When I’m in a relationship, I don’t manage to be faithful.
- The thought of being sexually attached to one person fills me with boredom.
- The thought of becoming sexually attached to a person scares me.
- I sometimes have sex with people who make me feel disgusted during or after sex.

Sexual risk behaviour

- I have already been infected with a sexually transmitted disease because I did not use adequate contraception during sex.

*Answer options: Yes; No; I do not know; Prefer not to say*

You see statements below about specific sexual activities and consequences. Please indicate how often these statements apply to you.

*Answer options: Never; Seldom; Occasionally; Sometimes; Often; Very often; Always; Prefer not to say*

- I have performed sexual acts that are not legally allowed.
- I have prostituted myself.
- I have performed sexual acts that harmed someone else physically or emotionally.
- I have had sex under the influence of illegal drugs.
- I am ashamed of my sexual activities.
- I feel guilty about my sexual activities.
- My sexual activities strongly impair important relationships with other people in my life.

Sexual dysfunction

You see statements below about specific sexual activities and consequences. Please indicate how often these statements apply to you.

*Answer options: Never; Seldom; Occasionally; Sometimes; Often; Very often; Always; Prefer not to say*

- I orgasm too quickly.
- I never orgasm.
- I need a long time before I orgasm.
- I do not experience sexual desire.
- I have erection problems/problems to get sexually aroused.
- I experience pain during sex.

Feelings of shame and guilt regarding masturbation

You can see below some statements about possible attitudes regarding masturbation. Please indicate whether and, if so, to what extent these statements apply to you.

*Answer options: Never; Seldom; Occasionally; Sometimes; Often; Very often; Always; Prefer not to say*

“After masturbation, …”

- I feel ashamed.
- I wonder what my parents might think of me.
- I feel like I was perverse/naughty.
- I do not feel comfortable.
- I wonder what other people (e.g., friends, colleagues) might think of me.
- I feel guilty.

**Question block 4: Personality traits**

You see below statements about specific personality aspects. Please estimate to what extent these statements apply to you.

*Answer options: Strongly disagree; Rather disagree; Partly agree; Rather agree; Strongly agree*

Impulsive personality tendencies

*Following the Impulsiveness Scale (Skala Impulsivität), German and English version by Kovaleva et al. [4].*

- I sometimes do things impulsively that I should not do.
- I sometimes do things to cheer myself up that I later regret.
- I usually think carefully before I act.
- I always bring to an end what I have started.
- I am willing to take risks.

Narcissistic personality tendencies

*Following the Narcissistic Personality Inventory, German version by von Collani [5].*

- It is easy for me to manipulate others.
- I like giving instructions to others.
- I will never be satisfied until I get everything that is due to me.
- I am more capable than other people.
- I can usually talk my way out of any unpleasant situations.
- I tend to brag when I get the opportunity.

Histrionic personality tendencies

*Following the Cognitive Profile of Histrionic Personality Disorder by Beck et al. [6].*

- I deserve the attention of the people around me in every case.
- I am interested in others as long as they show me attention and affection.
- I am afraid of being rejected.
- I need the admiration of others to be happy.
- I show my feelings in a particularly dramatic way to get what I want.

Depressive tendencies

*Following the Patient Health Questionnaire, PHQ-9, German version by Löwe et al. [7].*

- I have little interest or pleasure in doing things.
- I often feel hopeless.
- I often feel down/depressed.

Self-esteem

*Following the Self-Esteem Scale by Rosenberg [8].*

- On the whole, I am satisfied with myself.
- I feel that I have a number of good qualities.
- I feel I do not have much to be proud of.
- I certainly feel useless at times.
- I am able to do things as well as most other people.
- I take a positive attitude toward myself.

Loneliness

*Following the UCLA Loneliness Scale by Russel et al. [9].*

- There are people I feel close to.
- I can find companionship when I want it.
- *(Added item)* I often feel lonely.
- *(Added item)* I would describe myself as a loner.

Stresses in primary socialisation

*Following the Biographical Questionnaire for Alcohol-dependent People (Biografischer Fragebogen für Alkoholabghängige, BIFA-AL) by Bühler [10].*

- I have occasionally had the feeling that I do not belong in my family.
- My father always found fault with me about something or other.
- My parents were there for me when I had problems.
- I did not feel good at home because we did not have a good family life.
- *(Added item)* My mother always found fault with me about something or other.
- *(Added item)* I was often punished too harshly in my childhood/youth.

**Additional items**

*Answer options: Yes; No; Prefer not to say.*

- I have already received psychotherapeutic/psychiatric treatment.
- I am currently receiving psychotherapeutic/psychiatric treatment.
- I experienced harassment during my school years.
- I have experienced harassment in my adulthood.
- I was sexually abused when I was a child.
- I have been sexually abused in my adulthood.

**References**

1. Klein V, Rettenberger M, Boom KD, Briken P. Eine Validierungsstudie der deutschen Version des Hypersexual Behavior Inventory (HBI) [A validation study for the German version of the Hypersexual Behaviour Inventory]. *Psychother Psychosom Med Psychol*2014; **64**:136–40. doi:10.1055/s-0033-1357133.

2. Böthe B, Toth-Kiraly I, Zsila A, Griffiths MD, Demetrovics Z, Orosz G. The development of the Problematic Pornography Consumption Scale (PPCS). *J Sex Res* 2018; **55**(3): 395–406. doi:10.1080/00224499.2017.1291798.

3. Young C, Muehlenhard C. Attitude towards masturbation scale. In: Milhausen R, Sakaluk J, Fisher T, Davis C, Yarber W, editors. *Handbook of Sexuality-related Measures*. 4th ed. London: Routledge; 2020. p. 147–54.

4. Kovaleva A, Beierlein C, Kemper C, Rammstedt B. Die Skala Impulsives-Verhalten-8 (I-8) [Impulsivity scale 8]. *ZIS* 2014. doi: 10.6102/zis183

5. Von Collani G. Modifizierte deutsche Versionen des Narcissistic Personality Inventory (NIP-d) [Modified German version of Narcissistic Personality Inventory]. 2014.

6. Beck A, Freeman A, Pretzer J, Davis D, Fleming B, Orraviani R. *Kognitive Therapie der Persönlichkeitsstörungen [Cognitive therapy of personality disorders]*. Weinheim: Beltz; 1999.

7. Löwe B, Spitzer R, Zipfel S, Herzog W. PHQ-D - Gesundheitsfragebogen für Patienten [PHQ-D - Health questionnaire for patients]. 2002. https://www.klinikum.uni-heidelberg.de/fileadmin/Psychosomatische_Klinik/download/PHQ_Kurzanleitung1.pdf,

8. Rosenberg M. Self-Esteem Scale. 2014. doi: 10.6102/zis46

9. Russel D, Peplau LA, Cutrona CE. The revised UCLA Loneliness Scale: Concurrent and discriminant validity evidence. *J Pers Soc Psychol* 1980; **39**(3): 472–80.

10. Bühler KE. *Biographischer Fragebogen für Alkoholabhängige (BIFA-AL) [Biographical Questionnaire for Alcohol-dependent People]*. 2014. doi: 10.6102/zis88
